# Da-Chai-Hu Decoction Ameliorates High Fat Diet-Induced Nonalcoholic Fatty Liver Disease Through Remodeling the Gut Microbiota and Modulating the Serum Metabolism

**DOI:** 10.3389/fphar.2020.584090

**Published:** 2020-11-27

**Authors:** Huantian Cui, Yuting Li, Yuming Wang, Lulu Jin, Lu Yang, Li Wang, Jiabao Liao, Haoshuo Wang, Yanfei Peng, Zhaiyi Zhang, Hongwu Wang, Xiangguo Liu

**Affiliations:** ^1^ Shandong Provincial Key Laboratory of Animal Cell and Developmental Biology, School of Life Sciences, Shandong University, Qingdao, China; ^2^ Tianjin University of Traditional Chinese Medicine, Tianjin, China; ^3^ First Teaching Hospital of Tianjin University of Traditional Chinese Medicine, Tianjin, China; ^4^ Tianjin Second People’s Hospital, Tianjin, China; ^5^ Jiaxing Hospital of Traditional Chinese Medicine, Jiaxing, China

**Keywords:** nonalcoholic fatty liver disease, Da-Chai-Hu decoction, gut microbiota, untargeted metabolomics, correlation analysis

## Abstract

The dysbiosis in gut microbiota could affect host metabolism and contribute to the development of nonalcoholic fatty liver disease (NAFLD). Da-Chai-Hu decoction (DCH) has demonstrated protective effects on NAFLD, however, the exact mechanisms remain unclear. In this study, we established a NAFLD rat model using a high fat diet (HFD) and provided treatment with DCH. The changes in gut microbiota post DCH treatment were then investigated using 16S rRNA sequencing. Additionally, serum untargeted metabolomics were performed to examine the metabolic regulations of DCH on NAFLD. Our results showed that DCH treatment improved the dyslipidemia, insulin resistance (IR) and ameliorated pathological changes in NAFLD model rats. 16S rRNA sequencing and untargeted metabolomics showed significant dysfunction in gut microbiota community and serum metabolites in NAFLD model rats. DCH treatment restored the dysbiosis of gut microbiota and improved the dysfunction in serum metabolism. Correlation analysis indicated that the modulatory effects of DCH on the arachidonic acid (AA), glycine/serine/threonine, and glycerophospholipid metabolic pathways were related to alterations in the abundance of *Romboutsia*, *Bacteroides, Lactobacillus, Akkermansia*, *Lachnoclostridium* and *Enterobacteriaceae* in the gut microflora. In conclusion, our study revealed the ameliorative effects of DCH on NAFLD and indicated that DCH’s function on NAFLD may link to the improvement of the dysbiosis of gut microbiota and the modulation of the AA, glycerophospholipid, and glycine/serine/threonine metabolic pathways.

## Introduction

Accumulating numbers of studies have shown that the dysbiosis in gut microbiota could contribute to the development of nonalcoholic fatty liver disease (NAFLD) (Stefano et al., 2018). The composition of gut microbiota in NAFLD patients exhibited a distinct profile when compared to healthy controls (Li et al., 2018). Redundancy analysis (RDA) has also revealed significant correlations between fecal microbiota and related clinical outcomes, such as insulin resistance (IR) and dyslipidemia (Li et al., 2018). Additionally, researchers were able to induce NAFLD in germ free (GF) mice after they received a fecal transplantation from a high fat diet (HFD)-induced NAFLD mouse model (Porras et al., 2019). Modulation of gut microbiota using probiotics has shown beneficial effects on NAFLD mice. In one study, the oral treatment of *Lactobacillus rhamnosus GG* was able to protect mice from NAFLD by inhibiting the inflammatory response and improving the gut barrier function (Ritze et al., 2014).

Currently, lipid-lowering drugs, dietary therapy and exercise are the most commonly used treatments of NAFLD (Cao et al., 2020). However, lipid-lowering drugs, such as metformin and statins, exhibit numerous side-effects including gastrointestinal disorders (Scarpello et al., 1998), hepatotoxicity, and muscle aches (Bhardwaj and Chalasani, 2007). On the other hand, non-drug therapies like encouraging healthy habits of diet and exercise can be difficult for patients to maintain. The use of traditional Chinese medicine (TCM) has shown some protective effects on NAFLD and modulation of the gut microbiota has been demonstrated to be one of its key mechanisms (Cao et al., 2020). Da-Huang-Ze-Xie decoction has been shown to alleviate HFD-induced NAFLD model rats through altering the numbers of *Desulfovibrio*, *Escherichia/Shigella*, *Bacteroides*, *Oscillibacter* and *Butyricicoccus* in gut, improving gut permeability and inhibiting the activation of the Toll-like receptor 4 (TLR4) signaling pathway in liver (Jing et al., 2017). Qu-Shi-Hua-Yu decoction has been indicated to improve blood lipid levels and hepatic steatosis in NAFLD model rats through increasing the short chain fatty acid (SCFA)-producing gut microbiota (Yin et al., 2013).

Metabolomics could identify and quantify the levels of metabolites systematically and could be used to elucidate the pathogenesis of diseases and the mechanisms of drugs on metabolic levels (Su et al., 2018). Metabolites are metabolic byproduct biomolecules with low molecular weights that serve as signaling molecules and energy sources during various biological processes. Metabolite levels could be influenced by the gut microbiota and could directly reflect the current metabolic state of organs or cells (Tremaroli and Backhed, 2012). The combination of 16S rRNA sequencing and metabolomics could elucidate the mechanisms of Chinese herbal formulas via the interactions between gut microbiota and host metabolism. Combining the 16S rRNA sequencing with metabolomics analysis, Piao et al. demonstrated that Fu-Fang-Zhen-Zhu-Tiao-Zhi capsules exhibited anti-aging effects by increasing the SCFA-producing bacteria, decreasing the hydrogen sulfide-producing bacteria, and improving glucose-lipid metabolism (Piao et al., 2020). Kang-Shuai-Lao-Pian was found to affect the numbers of *Intestinimonas*, *Oscillibacter*, *Christensenellaceae_R-7_group*, and *Lachnoclostridium_UCG-010*, *Aliihoeflea* as well as regulate lysine, dipeptide, fatty acid and purine metabolism in obese mice (Gong et al., 2020).

Da-Chai-Hu decoction (DCH), composed of *Bupleurum chinense* DC., *Scutellariae baicalensis* Georgi, *Paeonia lactiflora* Pall., *Pinellia ternata* (Thunb.) Makino, *Citrus* × *aurantium* L., *Zingiber officinale* Roscoe, *Ziziphus jujuba* Mill. and *Rheum officinale* Baill., has been shown to exhibit beneficial effects on hyperlipidaemia and hypercholesterolemia (Yoshie et al., 2004; Iizuka et al., 2013; Fan and Miao 2014). Furthermore, it has been demonstrated that DCH could decrease the level of low density lipoprotein (LDL) in hypercholesterolemia animal models through up-regulating the expression of LDL-receptor in liver (Yoshie et al., 2004). However, the exact mechanisms of DCH on NAFLD remain unclear. To better understand these mechanisms, we established a NAFLD rat model using HFD and a DCH treatment regimen. The changes of gut microbiota post DCH treatment were investigated using 16S rRNA sequencing. In addition, untargeted metabolomics was performed to investigate the metabolic regulations of DCH on NAFLD. Understanding how DCH ameliorates NAFLD will provide clinicians with another valuable therapeutic tool and help unravel the link between Chinese herbal formulas, gut microflora, and patient outcomes.

## Materials and Methods

### Reagents

HFD (17.7% sucrose, 17.7% fructose, 19.4% protein and 40% fat) was obtained from Beijing Huafukang Bioscience Co., Ltd. (Beijing, China). Triglyceride (TG), total cholesterol (TC), aspartate aminotransferase (AST), alanine aminotransferase (ALT), superoxide dismutase (SOD), methane dicarboxylic aldehyde (MDA), and glutathione peroxidase (GSH-Px) assay kits test kits were obtained from Nanjing Jiancheng Biological Engineering Institute (Nanjing, China). Oil Red O Staining kit was purchased from Solarbio Biotechnology Co., Ltd. (Beijing, China). Rat insulin enzyme-linked immunosorbent assay (ELISA) kit was obtained from Multi Science Biotechnology Co., Ltd. (Hangzhou, China). Reference standards of paeoniflorin, baicalin, saikosaponin A, saikosaponin D, emodin, synephrine, succinic acid, 6-gingerol and oleanolic acid were obtained from Sichuan Weikeqi Biological Technology Co., Ltd. (Sichuan, China).

### Preparation of Da-Chai-Hu Decoction

The dosage of each drug in the DCH preparation used in this study was in accordance with the record in “Treatise on Febrile Diseases”, written by Zhang Zhongjing in 200 C.E.–210 C.E.. DCH contained: 12 g of *Bupleurum chinense* DC. (Tianjin traditional Chinese Medicine prepared pieces Co., Ltd, Tianjin, China, Batch number: 1907026), 9 g of *Scutellariae baicalensis* Georgi (Tianjin traditional Chinese Medicine prepared pieces Co., Ltd, Tianjin, China, Batch number: 1906002), 9 g of *Paeonia lactiflora* Pall. (Tianjin traditional Chinese Medicine prepared pieces Co., Ltd, Tianjin, China, Batch number: 1901016), 9 g *o*f *Pinellia ternata* (Thunb.) Makino (Tianjin traditional Chinese Medicine prepared pieces Co., Ltd, Tianjin, China, Batch number: 1812006), 9 g of *Citrus* × *aurantium* L. (Tianjin traditional Chinese Medicine prepared pieces Co., Ltd, Tianjin, China, Batch number: 1901016), 15 g of *Zingiber officinale* Roscoe (Tianjin traditional Chinese Medicine prepared pieces Co., Ltd, Tianjin, China, Batch number: 1903002), 12 g of *Ziziphus jujuba* Mill. (Tianjin traditional Chinese Medicine prepared pieces Co., Ltd, Tianjin, China, Batch number: 1907007), and 6 g of *Rheum officinale* Baill. (Tianjin traditional Chinese Medicine prepared pieces Co., Ltd, Tianjin, China, Batch number: 1902005). All herbs were authenticated by Pharmacist Li Wang in Department of Pharmacy of the Tianjin Second People’s Hospital. The above herbs were then soaked in 300 ml water for 30 min and decocted for 30 min to obtain the aqueous extract of DCH. The aqueous extract of DCH was filtered and concentrated to a density of 0.8 g crude herb/ml.

Quality control of DCH was performed using high performance liquid chromatography (HPLC; UltiMate 3,000, Thermo Scientific^™^, USA) coupled with mass spectrometer (MS; Q Exactive^™^, Thermo Scientific^™^, USA). The chromatographic conditions were as follows: The chromatographic column was an Eclipse Plus C_18_ RRHD column (2.1 × 100 mm, 1.8 µm). The column temperature was maintained at 40°C and the flow rate was 0.3 ml/min. 0.1% formic acid aqueous solution (A) and acetonitrile (B) were used as the mobile phases and the injection volume was 5 μl. The mobile phase conditions were: 0 min, 5% B; 1 min, 5% B; 9 min 80% B; 12 min 100% B; 14 min 100% B; 14.1 min 5% B; 16 min 5% B. A mass spectrometer equipped with an electrospray ionization (ESI) source was used for both positive and negative ionization scan modes (m/z ranges from 100 to 1,500). The detailed parameters of MS were: spray voltage of 3,500 V (positive mode) and 3,000 V (negative mode), capillary temperature at 320°C (both positive and negative modes), sheath gas flow rate of 30 arbitrary units (both positive and negative modes), and auxiliary gas flow rate of 10 arbitrary units (both positive and negative modes).

### Animals and Treatment

6-8 weeks old male Sprague-Dawley (SD) rats weighing 190–210 g, were purchased from Huafukang Animal Co., Ltd. (Beijing, China). They were acclimated in a controlled environment (12 h light/dark cycle, 21 ± 2°C with a relative humidity of 45 ± 10%) with *ad libitum* access to food and water. All animal experiments were approved by the Animal Ethics Committee at Tianjin University of Traditional Chinese Medicine.

After the acclimatization for 1 week, all animals were randomly divided into four groups (n = 10): control, model, positive control and DCH. Rats in the control group were fed with standard laboratory chow, while rats in the model, positive control, and DCH groups received HFD for 12 weeks to induce NAFLD (Li et al., 2020). The ingredients of standard chow and HFD were showed in Table 1. After 4 weeks of HFD feeding, rats in the positive control and DCH groups were orally treated with metformin (200 mg/kg rat weight) (Zheng et al., 2014) and DCH (8 g/kg rat weight), respectively, once per day for 8 weeks. Whereas, rats in the control and model groups received an equivalent volume of saline. At the end of 8 weeks of metformin and DCH treatment, rats were sacrificed and their livers weighed. Liver index was calculated based on the percentage of liver to body weight.

**TABLE 1:** Composition of chows used in current study.

		Cereal (%)	Sucrose (%)	Fructose (%)	Protein (%)	Fat(%)
Standard diet		59.40	—	—	20	4.80
HFD		—	17.70	17.70	19.40	40

Vegetable oil and lard were used as the sources of fat in the standard diet and HFD respectively.

### Serum Biochemical Markers Assay

Serum samples were collected at the end of 8 weeks of metformin and DCH treatment for biochemical analysis. Briefly, rats were anaesthetized and blood was harvested by a syringe from the aorta abdominalis. The blood was then centrifuged at 3,000 rpm for 15 min to isolate the serum. The levels of TG, TC, ALT, and AST in serum were analyzed according to the manufacturer’s instructions provided by Nanjing Jiancheng Biological Engineering Institute (Nanjing, China) and the absorbance value was detected using a microplate reader (Varioskan Flash, Thermo Fisher, Massachusetts, USA).

### H&E Staining

After euthanasia, the rat’s livers were immediately removed and fixed in 10% formalin, dehydrated, and embedded in paraffin wax. The tissues were then cut into 5 µm sections using a microtome (RM2125, Leica, Buffalo Grove, USA) and were subsequently stained with hematoxylin and eosin (H&E), as has been previously described (Cui et al., 2018). The pathological severities of steatosis, lobular inflammation, and hepatocyte ballooning were determined using the NAFLD activity score (NAS) as described in our previous publication (Cui et al., 2020).

### Oil Red O Staining

Livers were sectioned into 20 μm thick coronal sections using a microtome-cryostat (CM3050S, Leica, Buffalo Grove, USA). The sections were then stained with Oil Red O, following the manufacturer’s instructions. The staining of lipid drops by Oil Red O was quantified using Image J to obtain the integrated optical density (IOD). The mean optical density (MOD) was calculated based on the ratio of IOD to the sum area.

### Oral Glucose Tolerance Test (OGTT)

OGTT was conducted at the end of 8 weeks of metformin and DCH treatment as has been previously described (Cummings et al., 2011). Briefly, rats were fasted for 16 h and the levels of fasting blood glucose (FBG) were determined. Then, rats received 50% glucose solution (1 g/kg) intragastrically and the blood glucose levels were measured at 30, 60 and 120 min post glucose solution treatment. The area under the curve (AUC) of OGTT was then calculated.

Determination of Homeostatic Model Assessment of Insulin Resistance (HOMA-IR).

Rats were fasted as detailed above and the levels of fasting insulin (FINS) in the serum were measured using ELISA according to the manufacturer’s instructions (Multi Science Biotechnology Co., Ltd., China). Additionally, the level of FBG was measured. The HOMA-IR was calculated using the following formula: HOMA-IR = FBG (mmol/L) × FINS (μU/ml)/22.5.

### Analysis of Liver Biochemical Markers

0.1 g of liver tissues were immersed in 900 µl normal saline followed by ultrasonic trituration to obtain liver tissue homogenates. The homogenates were then centrifugated at 3,000 rpm for 15 min and their supernatants were used to measure the MDA level as well as the SOD and GSH-Px activities according to the manufacturer’s instructions provided by Nanjing Jiancheng Biological Engineering Institute (Nanjing, China).

### Fecal 16S rRNA Sequencing

At the end of 8 weeks of DCH treatment, feces from the control, model, and DCH groups were simultaneously obtained under sterile conditions in a laminar flow hood. Fecal total DNAs were extracted and their purities and concentrations were measured by agarose gel electrophoresis. The DNA samples were then diluted to 1 ng/μl and polymerase chain reaction (PCR) was conducted to amplify the V4 region of 16S rRNA of DNA samples using specific primers with the barcode (forward: GTGCCAGCMGCCGCGGTAA reverse: GGACTACHVGGGTWTCTAAT). The PCR amplification mixture of each sample consisted of 15 μl of Phusion® High-Fidelity PCR Master Mix (New England Biolabs), 0.2 μM of forward and reverse primers, and 10 ng of template DNA. The PCR products were then quantified by 2% agarose gel electrophoresis and purified using Qiagen Gel Extraction Kit (Qiagen, Germany). The sequencing libraries were generated using TruSeqRDNA PCR Free Sample Preparation Kit (Illumina, United States) and then sequenced on the NovaSeq6000 platform to generate paired-end reads.

### Data Analysis of Fecal 16S rRNA Sequencing

Paired-end reads obtained from 16S rRNA sequencing were assigned to samples, truncated by cutting off the barcode and primer sequence, and merged by the FLASH V1.2.7 analysis tool (http://ccb.jhu.edu/software/FLASH/) to obtain the raw tags. The raw tags were then filtered to generate the clean tags according to the QIIME V1.9.1 quality controlled process (http://qiime.org/scripts/split_libraries_fastq.html). The chimera sequences in clean tags were detected and removed to obtain the effective tags using the UCHIME algorithm (http://www.drive5.com/usearch/manual/uchime_algo.html). The sequences of effective tags with ≥97% similarity were then assigned to the same OTUs using the Uparse V7.0.1001 software (http://drive5.com/uparse/). Representative sequences for each OTU were then screened for further annotation via the Silva database (http://www.arb-silva.de/). The relative abundances of OTUs were normalized using a standard of sequence number corresponding to the sample with the least sequences. The normalized data were then used for alpha diversity and beta diversity analysis. The gene family abundance of 16S rRNA sequencing data was predicted according to the Phylogenetic Investigation of Communities by Reconstruction of Unobserved States database (PICRUSt).

### Untargeted Metabolomics Study

At the end of 8 weeks of DCH treatment, serum samples from the control, model and DCH groups were collected for metabolomics analysis. The changes in serum metabolites were screened using liquid chromatography–mass spectrometry (LC–MS) and the data were analyzed using a method described in our previous study (Cui et al., 2020).

### Correlation Analysis Between Physiological Data, Untargeted Metabolomics Study and 16S rRNA Sequencing

Spearman’s correlation analysis was conducted to analyze the relationship between physiological data (body weight, liver index, TG, TC, AST, ALT, AUC of OGTT, FINS, and HOMA-IR), differential serum metabolites, and gut microbiota at genus level in the control, model and DCH groups.

### Statistics

All data were reported as the mean ± standard deviation (mean ± SD) for the independent experiments. Statistical differences between the experimental groups were examined using the analysis of variance (ANOVA) and SPSS software, version 20.0. A *p*-value < 0.05 was considered statistically signiﬁcant. Curve ﬁtting was performed using the GraphPad Prism 5 software.

## Results

### Identification of Main Bioactive Compounds in Da-Chai-Hu Decoction by HPLC-MS Analysis

HPLC-MS analysis was conducted to investigate the chemical profiles of DCH. Synephrine, succinic acid, paeoniflorin, baicalin, saikosaponin A, 6-gingerol, saikosaponin D, emodin and oleanolic acid were used as the reference standards to validate the main bioactive compounds in DCH. The molecular formulas and chemical structures of these reference standards were shown in Supplementary Figure S1. The typical based peak intensity (BPI) chromatograms of DCH and the reference standards were analyzed in both positive and negative modes (Supplementary Figure S2) and their characteristic fragment ions were shown in Supplementary Table S1. Paeoniflorin in *Paeonia lactiflora* Pall., baicalin in *Scutellariae baicalensis* Georgi, saikosaponin A and saikosaponin D in *Bupleurum chinense* DC., emodin in *Rheum officinale* Baill., synephrine in *Citrus* × *aurantium* L., succinic acid in *Pinellia ternata* (Thunb.) Makino, 6-gingerol in *Zingiber officinale* Roscoe and oleanolic acid in *Ziziphus jujuba* Mill. were identified as the preeminent bioactive compounds in DCH.

### Effects of Da-Chai-Hu Decoction on Dyslipidemia, Liver Function, IR and Oxidative Stress in NAFLD Model Rats

**FIGURE 1:**
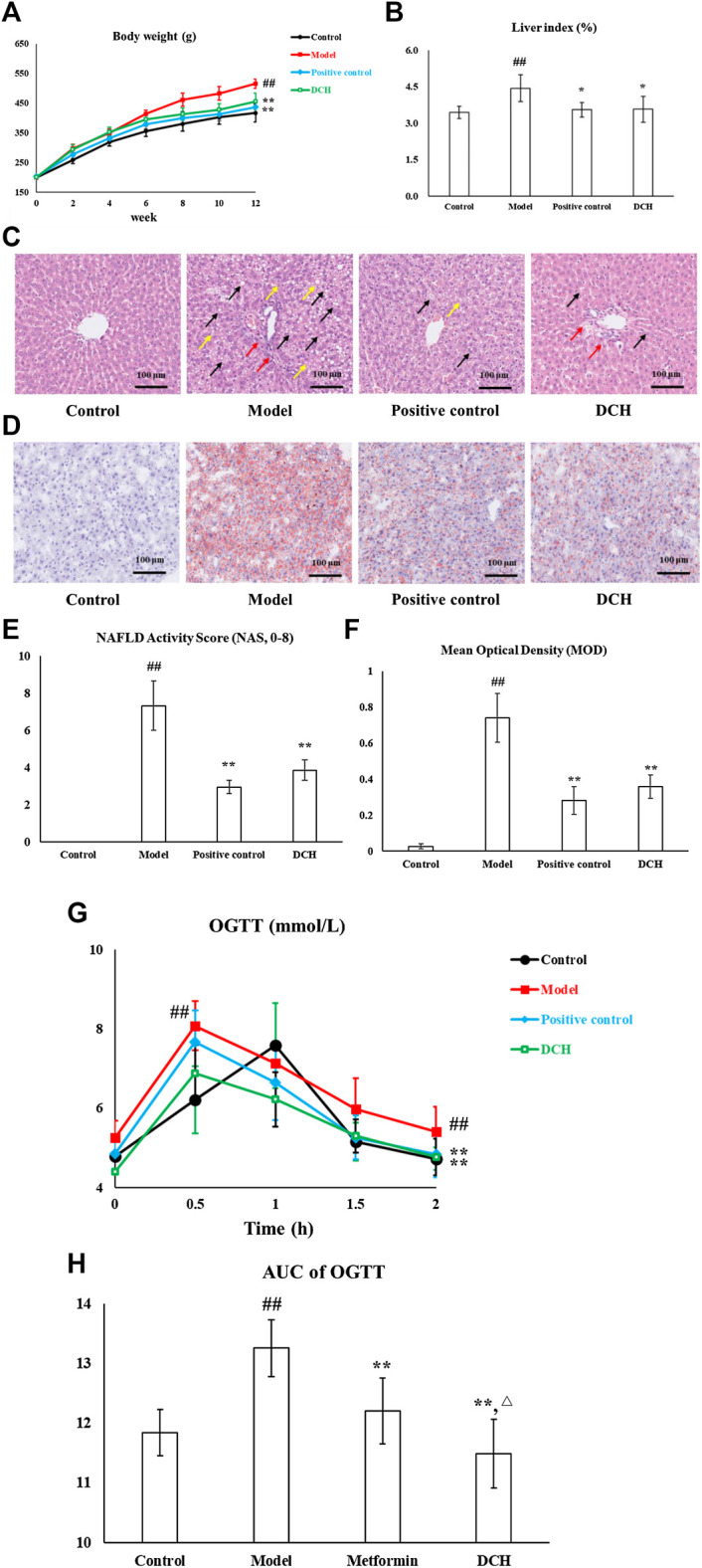
DCH treatment improved the hepatosteatosis, IR and oxidative stress in NAFLD model rats. **(A,B)** DCH treatment decreased the body weight and liver index in NAFLD model rats. **(C,E)** H&E staining showed that DCH treatment ameliorated the hepatic steatosis, hepatocytes ballooning, and lobular inflammation in the liver. Black arrows indicate the steatosis of hepatocytes, red arrows indicate lobular inflammation and yellow arrows indicate hepatocyte ballooning (×200 magnification). **(D,F)** Oil Red O staining shows that DCH treatment improved the lipid accumulation in the liver (200×). **(G,H)** The AUC of OGTT was decreased in NAFLD model rats after DCH treatment. Control, model, positive control and DCH (n = 10 per group) groups. Data are presented as the mean ± SD. ^##^: *p* < 0.01 as compared to the control group; *: *p* < 0.05 as compared to the model group; **: *p* < 0.01 as compared to the model group; ^△^: *p* < 0.05 as compared to the positive control group.

After 8 weeks of metformin and DCH treatment, the body weight (*p* < 0.01, Figure 1A), liver index (*p* < 0.01, Figure 1B) and serum levels of TG, TC, AST, and ALT (*p* < 0.01, respectively, Table 2) were significantly increased in model group compared with the control group. Whereas, metformin and DCH treatment significantly decreased the body weight (*p* < 0.01, respectively, Figure 1A), liver index (*p* < 0.05, respectively, Figure 1B), and serum levels of TG, TC, AST, and ALT (*p* < 0.01, respectively, Table 2) compared to the NAFLD model rats. There were no significant differences in body weight, liver index and levels of serum ALT and TG between the positive control and DCH groups (Table 2), however, the levels of serum TC and AST were significantly higher in DCH group compared with those in positive control group (*p* < 0.01, respectively, Table 2). Notable steatosis of hepatocytes accompanied by hepatocytes ballooning and lobular inflammation was revealed by H&E staining in the model group, while metformin and DCH treatment improved the hepatic steatosis, hepatocytes ballooning, and lobular inflammation (Figure 1C). Likewise, the NAS score was significantly higher in the model group compared with the control (*p* < 0.01, Figure 1E) and H&E staining of the positive control and DCH groups showed a lower NAS score compared with the model group (*p* < 0.01, respectively, Figure 1E). There were no significant differences in NAS between the positive control and DCH groups (Figure 1E). Oil Red O staining also confirmed hepatic steatosis and increased lipid deposition in the model group (*p* < 0.01, Figures 1D,F), whereas the numbers of lipid-loaded hepatocytes were significantly decreased in metformin and DCH treated rats (*p* < 0.01, respectively. Figures 1D,F). There were no significant differences in the numbers of lipid-loaded hepatocytes between the positive control and DCH groups (Figure 1F).

**TABLE 2:** Changes in blood lipid levels and liver enzymes after DCH treatment.

Group	TC (mmol/L)	TG (mmol/L)	ALT (IU/L)	AST (IU/L)
Control	1.35 ± 0.29	0.27 ± 0.04	44.25 ± 7.59	131.02 ± 16.54
Model	2.44 ± 0.39^##^	1.78 ± 0.24^##^	159.99 ± 20.73^##^	197.56 ± 23.00^##^
Positive control	1.51 ± 0.07**	1.00 ± 0.64**	109.81 ± 17.69**	139.19 ± 12.71**
DCH	1.80 ± 0.13**^,△△^	1.05 ± 0.28**	128.01 ± 15.59**	166.84 ± 18.15**^,△△^

Control, model, positive control and DCH (n = 10 per group) groups. Data are presented as the mean ± SD. ^##^: *p* < 0.01 as compared to the control group; **: *p* < 0.01 as compared to the model group; ^△△^: *p* < 0.01 as compared to the positive control group.

IR is also an important clinical outcome of NAFLD and as such we measured the effects of DCH on IR. The AUC of OGTT was significantly increased in model group compared with the control group (*p* < 0.01, Figures 1G,H), whereas metformin and DCH treated rats displayed a lower AUC of OGTT compared with the NAFLD model rats (*p* < 0.01, respectively, Figures 1G,H). The AUC of OGTT was lower in the DCH group compared with the positive group (*p* < 0.05, Figures 1G,H). Additionally, the levels of FINS and HOMA-IR were higher in the model group compared with the control group (*p* < 0.01, Table 3) and were lower in the positive control and DCH groups compared to the model group (*p* < 0.01, respectively, Table 3). There were no significant differences in FINS and HOMA-IR between the positive control and DCH groups (Table 3).

**TABLE 3:** Levels of FINS and HOMA-IR after DCH treatment.

Group	FINS (μU/ml)	HOMA-IR
Control	7.6 ± 1.33	1.14 ± 0.23
Model	16.46 ± 2.07^##^	3.56 ± 0.55^##^
Positive control	11.43 ± 1.41**	2.08 ± 0.48**
DCH	13.53 ± 1.65**	2.42 ± 0.32**

Control, model, positive control and DCH (n = 10 per group) groups. Data are presented as the mean ± SD. ^##^: *p* < 0.01 as compared to the control group; **: *p* < 0.01 as compared to the model group.

We also investigated the anti-oxidative effects of DCH on NAFLD. Our results showed significantly lower activities of SOD, GSH-Px, and higher levels of MDA in the NAFLD model rats compared to rats in the control group (*p* < 0.01, respectively, Table 4). Compared with the model group, the activities of SOD (*p* < 0.05, respectively, Table 4) and GSH-Px (*p* < 0.01, respectively, Table 4) were increased and the level of MDA (*p* < 0.01, respectively, Table 4) was decreased in the positive control and DCH groups.There were no significant differences in SOD, GSH-Px and MDA between the positive control and DCH groups (Table 4).

**TABLE 4:** The activities of SOD and GSH-Px and the levels of MDA in rat liver homogenate after DCH treatment.

Group	SOD (U/mgprot)	MDA (nmol/mgprot)	GSH-Px (U/mgprot)
Control	41.72 ± 4.39	2.78 ± 0.48	171.44 ± 3.82
Model	32.20 ± 4.94^##^	15.96 ± 2.01^##^	126.82 ± 9.87^##^
Positive control	39.35 ± 4.19*	7.86 ± 1.07**	159.30 ± 11.93**
DCH	38.62 ± 3.58*	8.81 ± 0.95**	154.69 ± 10.82**

Control, model, positive control and DCH (n = 10 per group) groups. Data are presented as the mean ± SD. ^##^: *p* < 0.01 as compared to the control group; *: *p* < 0.05 as compared to the model group; **: *p* < 0.01 as compared to the model group.

### Modulatory Effects of Da-Chai-Hu Decoction on Gut Microbiota in NAFLD Model Rats

**FIGURE 2:**
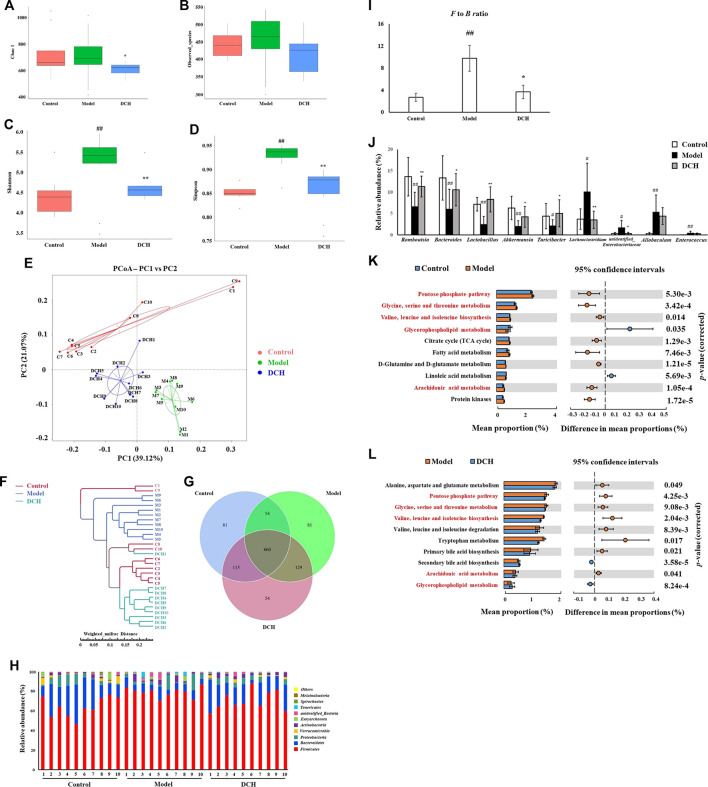
DCH treatment affected the gut microbiota community in NAFLD model rats. **(A,B)** There were no significant differences in Chao1 index and observed species number in each group. **(C,D)** Shannon and Simpson indexes were lower in DCH group than that in the model group. **(E,F)** PCoA and system clustering tree showed more similar beta diversity between DCH and control groups than that between the model and control groups. **(G)** The different numbers of OTUs were visualized in Venn diagram. **(H,I)** At the phylum level, DCH treatment decreased the *F* to *B* ratio in NAFLD model rats. **(J)** At the genus level, DCH treatment affected the relative abundances of *Romboutsia, Bacteroides, Lactobacillus*, *Akkermansia*, *Turicibacter, Lachnoclostridium* and *unidentified_Enterobacteriaceae* in NAFLD model rats. **(K,L)** The differential metabolic pathways (written in red) of DCH on NAFLD were predicted using PICRUSt analysis based on the 16S rRNA sequencing data. Control, model and DCH (n = 10 per group) groups. Data are presented as the mean ± SD. ^#^: *p* < 0.05 as compared to the control group; ^##^: *p* < 0.01 as compared to the control group; *: *p* < 0.05 as compared to the model group; **: *p* < 0.01 as compared to the model group.

16S rRNA sequencing was performed to detect gut microbiota changes in NAFLD model rats post DCH treatment. Overall, 2,196,489 useable reads and 1,174 OTUs were obtained from 30 samples. There were no significant differences in Chao1 index and observed species number between control and model groups, indicating that the richness of gut microbiota community was not changed after HFD treatment (Figures 2A,B). The Chao1 index was lower in DCH group than that in the model group, whereas there was no significant differences in observed species number between model and DCH groups (Figures 2A,B). The Shannon and Simpson indexes were higher in the model group compared with the control group and were lower in DCH group compared with the model group, indicating that the alpha diversity of gut microbiota community was increased in the model group compared with the control group and DCH treatment decreased the alpha diversity of gut microbiota community in NAFLD model rats (Figures 2C,D). Principal co-ordinates analysis (PCoA) showed significant variations of gut microbiota in each group, with a shorter distance between the control and DCH groups than between the model and DCH groups (Figure 2E). Likewise, system clustering tree analysis indicated that the distance from the control group to the DCH group was closer than either to the model (Figure 2F).

We further investigated the changes in the relative abundances of gut microbiota species. A venn diagram of the three groups demonstrated that 660 OTUs overlapped among all groups; 714 OTUs were present in the control and model groups; 775 in the control and DCH groups; and 789 in the model and DCH groups (Figure 2G). At the phylum level, *Firmicutes* and *Bacteroidetes* were the most abundant phyla among all samples (Figure 2H). The *Firmicutes* to *Bacteroidetes* (*F* to *B*) ratio was higher in the model group than in the control group (*p* < 0.01, Figure 2I), whereupon the *F* to *B* ratio was decreased after DCH treatment (*p* < 0.05, Figure 2I). At the genus level, the relative abundances of *Romboutsia, Bacteroides, Lactobacillus*, *Akkermansia*, and *Turicibacter* (*p* < 0.01, *p* < 0.01, *p* < 0.01, *p* < 0.01 and *p* < 0.05, respectively, Figure 2J) were signifiacntly lower and the relative abundances of *Lachnoclostridium, unidentified_Enterobacteriaceae, Allobaculum*, and *Enterococcus* (*p* < 0.05, *p* < 0.05, *p* < 0.01 and *p* < 0.01, respectively, Figure 2J) were significantly higher in the model group than that in the control group. Compared with the model group, DCH treatment significantly increased the relative abundances of *Romboutsia, Bacteroides, Lactobacillus*, *Akkermansia*, and *Turicibacter* (*p* < 0.01, *p* < 0.05, *p* < 0.01, *p* < 0.05 and *p* < 0.05, respectively, Figure 2J) and significantly decreased the relative abundances of *Lachnoclostridium* and *unidentified_Enterobacteriaceae* (*p* < 0.01 and *p* < 0.05, respectively, Figure 2J). Additionally, we predicted the possible pathways related to the differential gut microbiota at the genus level by PICRUSt analysis. The top 10 terms of metabolic pathways with the highest proportion and a *p*-value < 0.05 were listed in Figure 2K (control group vs. model group) and Figure 2L (model group vs. DCH group). Proportions of metabolic pathways that were increased in the model group but decreased in DCH group, or vice versa, were considered as differential pathways. The abundances of pentose phosphate, glycine/serine/threonine and arachidonic acid (AA) metabolic pathways as well as the valine, leucine and isoleucine biosynthesis pathways were all increased in the model group compared with the control group (Figure 2K). Conversely, the abundance of glycerophospholipid metabolism pathway was decreased in model group compared with the control group (Figure 2K). For the DCH group, the abundances of pentose phosphate, glycine/serine/threonine, and AA metabolic pathways along with the valine, leucine and isoleucine biosynthesis pathways were lower than the model group and the abundance of glycerophospholipid metabolism pathway was higher (Figure 2L).

### Effects of Da-Chai-Hu Decoction on Serum Metabolism in NAFLD Model Rats

**FIGURE 3:**
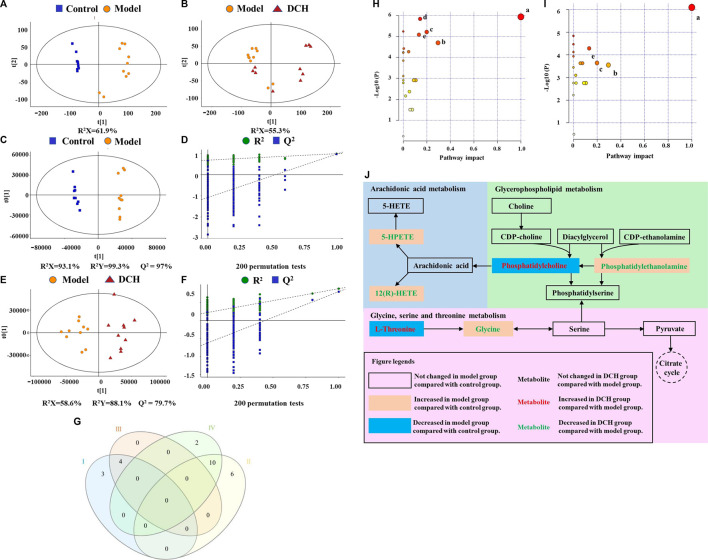
DCH treatment regulated the serum metabolites in NAFLD model rats. **(A,B)** Scores plots of PCA between the control and model groups and the model and DCH groups. **(C,D)** Scores plots of OPLS-DA between the control and model groups and the corresponding coefficient of loading plots. **(E,F)** Scores plots of OPLS-DA between the model and DCH groups and the corresponding coefficient of loading plots. **(G)** Numbers of differential metabolites between the control and model groups and the model and DCH groups (Venn diagram). **Ⅰ**: Decreased levels in the model group as compared to the control group; **Ⅱ:** Elevated levels in the model group as compared to the control group; **Ⅲ:** Elevated levels in the DCH group as compared to the model group; **Ⅳ:** Decreased levels in the DCH group as compared to the model group; **(H,I)** Summary of pathway analysis of serum samples between control and model groups and between model and DCH groups. **(A)** Linoleic acid metabolism; **(B)** Glycine, serine and threonine metabolism; **(C)** Glycerophospholipid metabolism; **(D)** Tryptophan metabolism; **(E)** AA metabolism. **(J)** Schematic of AA, glycerophospholipid, and glycine/serine/threonine metabolism pathways in NAFLD rats after DCH treatment. Control, model and DCH (n = 10 per group) groups.

The changes of metabolites in serum were investigated using untargeted metabolomics. According to the principle component analysis (PCA) model, a clear group separation could be observed between the control and model groups, while the distinction between the model and DCH groups was unclear (Figures 3A,B). Therefore, we performed orthogonal partial least squares discriminant analysis (OPLS-DA) to further visualize the metabolic alterations occurring between the control group and model group as well as between the model group and the DCH group. The OPLS-DA models showed significant distinctions of metabolomic data between the control group and the model group as well as between the model group and the DCH group (Figures 3C,E). The over-fitting in the OPLS-DA model was controlled using seven-round cross validation and 200 repetitions of RPT based on the R^2^ and Q^2^ values. The R^2^ and Q^2^ values of OPLS-DA model in the comparison of control and model groups were 0.686 and −1.08, respectively. The R^2^ and Q^2^ values in the OPLS-DA model comparing the model group with the DCH group were 0.22 and −0.624, respectively. These results indicated that the OPLS-DA models were robust (Figures 3D,F). Metabolites with a VIP >1 and *p* < 0.05 between control and model groups or between DCH and model groups were considered to be differential metabolites. The numbers of differential metabolites between the control group and the model group as well as between the model group and the DCH group were visualized in Figure 3G. Compared with the control group, the levels of phosphatidylcholine (PC), rumenic acid, linoleic acid, eicosapentaenoic acid (EPA), L-threonine, gluconic acid, and lacto-N-tetraose were decreased and the levels of L-proline, L-lysine, L-isoleucine, L-valine, L-arginine, L-leucine, glycocholic acid, uric acid, creatinine, stearic acid, ursodeoxycholic acid, phosphatidylethanolamine (PE), L-tryptophan, 12(R)-HETE, 5-HPETE, and glycine were increased in the model group. For the DCH group compared with the model group: the levels of linoleic acid, PC, L-threonine, and rumenic acid were increased and the levels of L-isoleucine, L-valine, L-arginine, L-leucine, stearic acid, indoleacrylic acid, THTC, 12(R)-HETE, 5-HPETE, glycine, uric acid, and PE were decreased (Table 5).

**TABLE 5:** The differential metabolites in serum after DCH treatment.

No	Rt (min)	m/z	Formula	Metabolites	VIP		FC		Trend		Pathway
					M vs. C	D vs. M	M vs. C	D vs. M	M vs. C	D vs. M	
1	1.06	116.0703	C_5_H_9_NO_2_	L-Proline	2.43	0.93	1.86	0.81	↑##	↓	—
2	0.92	120.0651	C_4_H_9_NO_3_	L-Threonine	2.18	3.55	0.33	3.01	↓##	↑**	b
3	0.76	147.1121	C_6_H_14_N_2_O_2_	L-Lysine	1.38	0.69	3.24	0.92	↑##	↓	—
4	2.69	132.1014	C_6_H_13_NO_2_	L-Isoleucine	2.94	1.49	1.98	0.67	↑##	↓*	—
5	1.43	118.0860	C_5_H_11_NO_2_	L-Valine	2.79	2.48	2.38	0.52	↑##	↓**	—
6	0.71	281.2460	C_18_H_32_O_2_	Linoleic acid	1.28	1.13	0.63	1.63	↓#	↑**	a
7	0.88	175.1181	C_6_H_14_N_4_O_2_	L-Arginine	2.77	1.31	1.94	0.66	↑##	↓**	—
8	2.92	132.1014	C_6_H_13_NO_2_	L-Leucine	3.89	2.08	1.85	0.63	↑##	↓**	—
9	10.42	301.2171	C_20_H_30_O_2_	Eicosapentaenoic acid	1.06	2.73	0.60	1.00	↓#	—	—
10	8.80	464.3011	C_26_H_43_NO_6_	Glycocholic acid	1.73	0.43	2.24	0.66	↑##	↓	—
11	2.30	169.0349	C_5_H_4_N_4_O_3_	Uric acid	2.37	1.16	1.81	0.65	↑##	↓**	—
12	0.95	114.0659	C_4_H_7_N_3_O	Creatinine	2.01	0.77	3.29	0.75	↑##	↓	—
13	14.29	283.2639	C_18_H_36_O_2_	Stearic acid	2.30	1.88	2.04	0.53	↑##	↓**	—
14	9.52	391.2857	C_24_H_40_O_4_	Ursodeoxycholic acid	1.66	0.64	2.25	0.75	↑##	↓	—
15	9.83	241.0560	C_6_H_12_O_7_	Gluconic acid	1.23	0.89	0.63	0.99	↓##	↓	—
16	10.85	730.2405	C_26_H_45_NO_21_	Lacto-N-tetraose	1.12	1.31	0.87	0.92	↓#	↓	—
17	0.83	940.8140	C_56_H_112_NO_8_P	Phosphatidylcholine	2.30	2.02	0.49	1.93	↓##	↑**	c, e
18	10.99	279.2327	C_18_H_32_O_2_	Rumenic acid	5.93	3.92	0.50	1.86	↓##	↑**	—
19	10.58	766.4845	C_45_H_70_NO_8_P	Phosphatidylethanolamine	1.33	2.88	1.31	0.79	↑#	↓*	c
20	5.77	203.0816	C_11_H_12_N_2_O_2_	L-Tryptophan	1.52	0.67	1.88	0.85	↑##	↓	d
21	9.43	319.2273	C_20_H_32_O_3_	12(R)-HETE	4.68	2.49	1.83	0.46	↑##	↓**	e
22	9.54	317.2116	C_20_H_32_O_4_	5-HPETE	1.20	2.13	1.54	0.50	↑#	↓**	e
23	0.83	151.0721	C_2_H_5_NO_2_	Glycine	1.12	2.02	1.56	0.47	↑#	↓**	b
24	5.75	188.0697	C_11_H_9_NO_2_	Indoleacrylic acid	0.86	3.33	1.22	0.47	↑	↓*	—
25	1.72	150.0576	C_5_H_8_O_2_S	THTC	0.64	1.32	1.39	0.44	↑	↓*	—

Control, model, positive control and DCH (n = 10 per group) groups. ^#^, *p* < 0.05 as compared to the control group; ^##^, *p* < 0.01 as compared to the control group; *, *p* < 0.05 as compared to the model group; **, *p* < 0.01 as compared to the model group; ↑, content increased; ↓, content decreased; vs., versus; C, control group; M, model group; D, DCH group. (a) Linoleic acid metabolism. (b) Glycine, serine and threonine metabolism. (c) Glycerophospholipid metabolism. (d) Tryptophan metabolism. (e) AA metabolism.

In addition, differential metabolites with a fold change (FC) greater than 1.2 or a FC of less than 0.8 were analyzed using MetaboAnalyst software to screen for significant metabolic pathways (*p* < 0.05, impact value >0.10). Tryptophan, AA, linoleic acid, glycerophospholipid, and glycine/serine/threonine metabolisms were identified to be significant metabolic pathways between the control and model groups (Figure 3H). Between the model and DCH groups, AA, linoleic acid, glycerophospholipid, and glycine/serine/threonine metabolism were identified to be significant metabolic pathways (Figure 3I). The same pathways obtained from both PICRUSt analysis of 16S rRNA sequencing and pathway analysis of untargeted metabolomics, including AA, glycerophospholipid, and glycine/serine/threonine metabolic pathways, were visualized in Figure 3J and discussed in detail.

### Correlation Analysis of Physiological Data, Untargeted Metabolomics and Gut Microbiota

**FIGURE 4:**
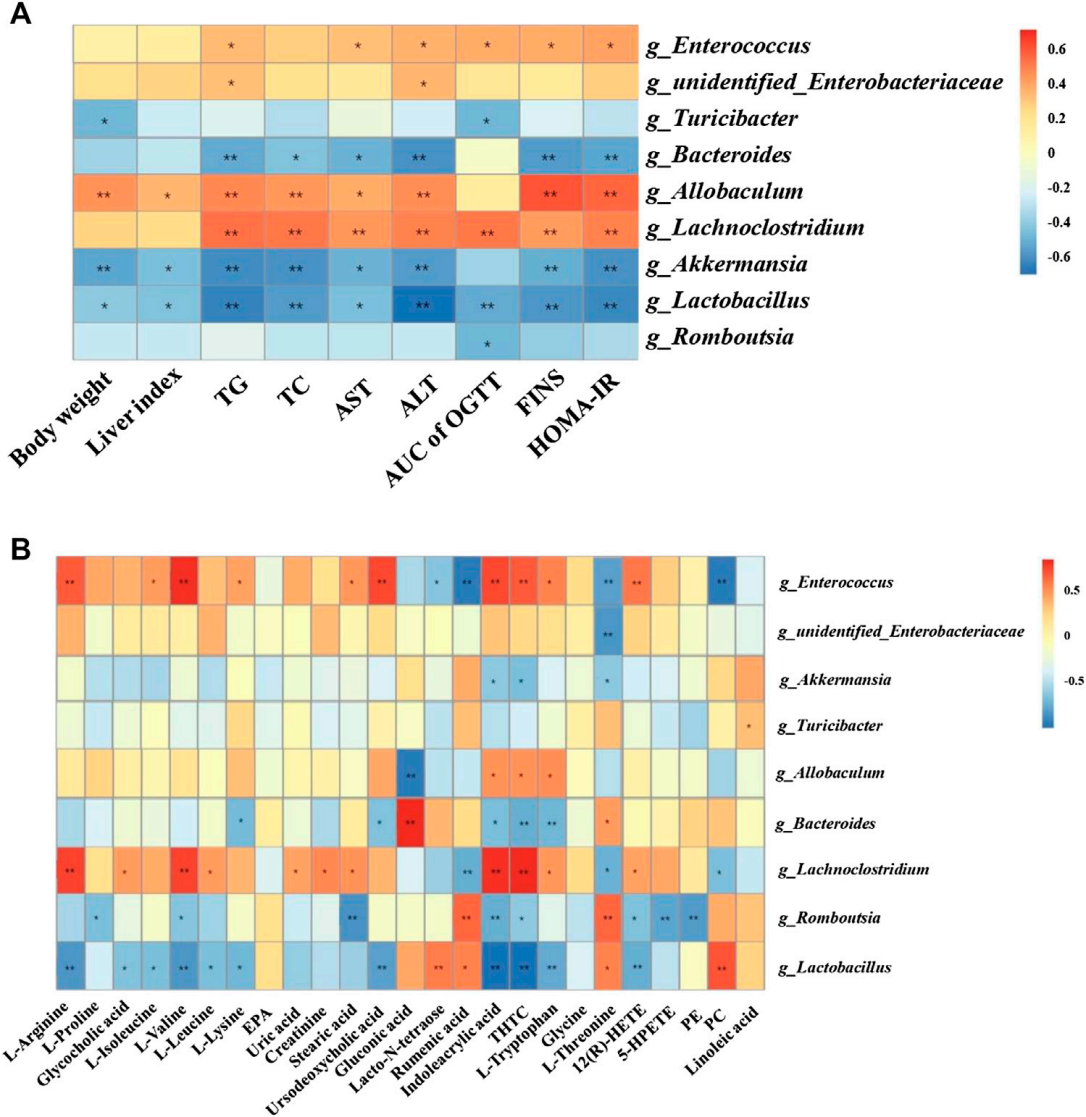
Correlation analysis of pathological indices, untargeted metabolomics and 16S rRNA sequencing. Correlations between physiological indices and gut microbiota **(A)** and between untargeted metabolomics and gut microbiota **(B)** were analyzed using spearman’s analysis (heatmap). X-axis represents the physiological indices **(A)** and differential metabolites in the serum **(B)**. Y-axis represents the gut microbiota with differential abundance. The colors of grids represent the correlation analysis value of spearman’s correlation analysis. Grids in red indicate positive correlations (correlation analysis value more than 0.1), while grids in blue indicate negative correlations (correlation analysis value less than −0.1). Color coding scale indicates the correlation analysis value from heatmap, the deeper red or blue indicates higher correlation values. *: *p* < 0.05 between physiological indices and differential gut microbiota **(A)**, or between differential serum metabolites and gut microbiota **(B)**. **: *p* < 0.01 between physiological indices and differential gut microbiota **(A)**, or between differential serum metabolites and gut microbiota **(B)**.

As shown in Figure 4A, *Lactobacillus* and *Romboutsia* have shown significant negative correlations with the pathological changes in NAFLD rat models, whereas *Enterococcus*, *Allobaculum,* and *Lachnoclostridium* have shown positive correlations with the pathological changes in NAFLD rat models.

Additionally, *Lactobacillus* showed positive correlations with PC, rumenic acid, and lacto-N-tetraose and negative correlations with the majority of the amino acids and metabolites related to AA metabolism. *Enterococcus* and *Lachnoclostridium* showed positive correlations with most of the amino acid metabolites (Figure 4B).

## Discussion

In this study, NAFLD rat model was induced using HFD. Consistent with the previous studies (Li et al., 2020), rats incurred remarkable disorders in serum levels of TG, TC, ALT and AST after HFD feeding. Pathological studies also indicated clear steatosis and cellular damage of hepatocytes in the model group. DCH treatment improved dyslipidemia and ameliorated pathological changes in the liver. Due to the close relationship between IR and NAFLD (Reenam et al., 2019), we also studied the effects of DCH on IR. Our results showed decreased levels of FINS, HOMA-IR and AUC of OGTT following DCH treatment, indicating that DCH could alleviate IR in NAFLD model rats. Metformin, which has been widely used as the positive control in both NAFLD and IR studies, was served as the positive control for our study (Zheng et al., 2014; Xie et al., 2019). Clinical studies have shown that treatment of metformin for 48 days could decrease body weight and improve the serum levels of ALT in NAFLD and NASH patients (Loomba et al., 2009). Meta-analysis of clinical trials has also indicated that metformin could improve liver function, HOMA-IR, and body mass index (BMI) in NAFLD patients (Li et al., 2013). Although our results showed higher serum levels of TC and AST in DCH treated rats compared with those treated with metformin, a lower AUC of OGTT was observed in DCH group when compared with the metformin treated rats. Furthermore, there were no significant differences in body weight, liver index, FINS, HOMA-IR, and levels of serum TG and ALT between DCH and metformin treated rats. The minimal difference between the two therapeutic test groups suggested that DCH had the potential to serve as alternative treatment to metformin on NAFLD and IR.

NAFLD could impact the body in accordance with the two-hit hypothesis proposed by Christopher Day and Oliver James. In the first hit, excessive lipid contents accumulating in the liver triggered the dysfunction of lipid metabolism. This was followed by the second hit, where oxidative stress and lipid peroxidation caused by the dysfunction of lipid metabolism, induce cellular damage of hepatocytes (Chen et al., 2008). Our results demonstrated significant anti-oxidative effects for DCH on NAFLD model rats, inducing increased activities of SOD and GSH-Px and a decreased level of MDA. The accumulation of lipids in hepatocytes triggered the disorder of fatty acid oxidation and induced the production of reactive oxygen species (ROS). Excessive ROS could induce the peroxidation of unsaturated fatty acid to generate MDA and impair the function of mitochondria (Vander Heiden et al., 1997). SOD and GSH-Px are anti-oxidative enzymes, playing key roles in the elimination of ROS (El-Din et al., 2014). Increasing the activities of SOD and GSH-Px inhibited oxidative stress and could improve liver function in NAFLD patients (Cui et al., 2020).

In addition, we conducted 16S rRNA sequencing of fecal total DNA to investigate the changes in microbiological composition following DCH treatment. Our results showed a higher alpha diversity in the gut microbiota in the model group compared with the control group, whereas, the alpha diversity of the gut microbiota was decreased after DCH treatment. Our findings were in agreement with the studies from other researchers that HFD treatment could cause a higher alpha diversity of gut microbiota community in the animal model of NAFLD (Wu et al., 2018). PCoA and system clustering tree analysis revealed that HFD treatment affected the beta diversity of gut microbiota, conversely, the beta diversities in DCH treated rats showed a greater similarity to rats in the control group than those in the model group. The ratio between *F* to *B* is closely related to dyslipidemia (Abdallah et al., 2011). Compared to healthy controls, patients with dyslipidemia showed a significant increase in the relative abundance of *Firmicutes* and a remarkable decrease of *Bacteroidetes*: an increase in *F* to *B* ratio (Abdallah et al., 2011; Kasai et al., 2015). Decreasing the *F* to *B* ratio in patients had achieved a better clinical outcome for dyslipidemia (Xu et al., 2017). Consistent with these observations, our results indicated a substantial increase in the *F* to *B* ratio in model group, that was decreased after DCH treatment. The relative abundances of *Romboutsia*, *Bacteroides, Lactobacillus, Akkermansia*, and *Turicibacter* were all increased post DCH treatment. *Bacteroides* are members in *Bacteroidetes* phylum and produce short chain fatty acids (SCFAs) through the fermentation of dietary fiber (Amabebe et al., 2020). SCFAs could be released into circulation and have been shown to help maintain the homeostasis of lipid metabolism in the liver (Nazli et al., 2004). *Lactobacillus* is a widely accepted probiotic and has used to treat dyslipidemia (Kadooka et al., 2010). *Akkermansia* has been reported to be negatively associated with inflammatory diseases and metabolic disorders (Muriel et al., 2017). In one study, supplementation with *Akkermansia* significantly improved gut barrier function, dyslipidemia, and IR in HFD-treated mice (Amandine et al., 2013). *Turicibacter* and *Romboutsia* have also been demonstrated to play beneficial roles in dyslipidemia (Kuda et al., 2017). Likewise in our study, spearman’s analysis showed negative correlations for *Romboutsia*, *Bacteroides, Lactobacillus, Akkermansia*, and *Turicibacter* with body weight and biochemical markers of dyslipidemia, liver injury, and IR. Our current analysis, in concert with previous studies, indicated that one of the mechanisms for the ameliorative effects of DCH on NAFLD might be increasing the abundance of *Romboutsia*, *Bacteroides, Lactobacillus, Akkermansia*, and *Turicibacter.* In addition to increasing the relative abundance of positive gut microbiota, the relative abundances of *Lachnoclostridium* and *Enterobacteriaceae* were decreased post DCH treatment. Studies have shown that the abundance *Enterobacteriaceae* is increased in NAFLD model rats and that decreasing the abundance the *Enterobacteriaceae* in gut could improve NAFLD prognosis (Liang et al., 2018; Liang et al., 2018). *Lachnoclostridium* has been recently demonstrated to be associated with the progression of colorectal cancer (Liang et al., 2019). However, the role of *Lachnoclostridium* plays in NAFLD remains unclear. Our spearman’s analysis showed positive correlations of *Enterococcus*, *Allobaculum*, *Lachnoclostridium*, and *Enterobacteriaceae* with the pathological changes of NAFLD. Decreasing the abundance of *Lachnoclostridium* and *Enterobacteriaceae* might be another ameliorative mechanism of DCH on NAFLD, although the impact of *Lachnoclostridium* on NAFLD needs to be further investigated to validate this supposition*.* Metabolic pathways related to the altered gut microbiota were also predicted using PICRUSt analysis. DCH treatment reversed the disorders in pentose phosphate, glycine/serine/threonine, AA and glycerophospholipid metabolic pathways in NAFLD model rats.

OPLS-DA of serum untargeted metabolomics showed the different metabolic profiles of the control, model and DCH groups, indicating that DCH could affect the metabolic profiles in HFD-induced NAFLD model rats. Pathway analysis of differential metabolites using MetaboAnalyst software showed that tryptophan, AA, linoleic acid, glycerophospholipid, and glycine/serine/threonine metabolic pathways were altered after HFD treatment and DCH could affect the AA, linoleic acid, glycerophospholipid, and glycine/serine/threonine metabolic pathways in NAFLD model rats. In particular, AA, glycerophospholipid, and glycine/serine/threonine metabolic pathways were identified in both PICRUSt analysis of gut microbiota studies and pathway analysis of untargeted metabolomics. Therefore, we hypothesized that DCH could regulate the disorders in AA, glycerophospholipid, and glycine/serine/threonine metabolic pathways through improving the disbiosis in gut microbiota.

### AA Metabolism

The dysfunction of AA metabolism has been previously implicated in the course of metabolic disorder and inflammatory diseases (Sonnweber et al., 2018). Our results showed that the levels of 12(R)-HETE and 5-HPETE, which are metabolic products of AA, were increased in NAFLD model rats. Whereupon, DCH treatment decreased the levels of 12(R)-HETE and 5-HPETE. The serum levels of 12(R)-HETE have been positively correlated to the severity of NAFLD (Maciejewska et al., 2015). A possible mechanism of action is that increased levels of 12(R)-HETE and 5-HPETE induce the inflammatory response by recruiting neutrophil cells (Han and Corey, 2000). Dietary intervention has been shown to decrease the levels of 12-HETE and ameliorate hepatic steatosis in NAFLD patients (Dominika et al., 2015). Furthermore, negative correlations between *Lactobacillus*, *Romboutsia,* and 12(R)-HETE, 5-HPETE levels were found by our spearman’s analysis. Likewise, other studies showed that excessive dietary polyunsaturated fatty acids, such as linoleic acid (LA), could augment the adipose inflammatory response in obese mice through inducing AA metabolism. Colonization of *Lactobacillus* could inhibite AA metabolism to ameliorate adipose inflammatory response and obesity through converting excessive dietary LA to 10-hydroxy-cis-12-octadecenoic acid (HYA) (Miyamoto et al., 2019). Up to now, *Romboutsia* has not been demonstrated to associate with AA metabolism. The detailed effects and mechanisms of *Romboutsia* on AA metabolism still need to be further investigated.

### Glycerophospholipid Metabolism

The dysfunction of glycerophospholipid metabolism has been shown to disturb the energy metabolism of hepatocytes (Mesens et al., 2012). Our results showed that the level of PC was decreased and the level of PE was increased in NAFLD model rats, with the inverse effect following DCH treatment. PCs comprise 40–50% of total phospholipids in mammalian cells (Vander Veen et al., 2017). The generation of PC includes two avenues. Choline is transformed into CDP-choline, an intermediate product in PC generation, and then CDP-choline interacts with diacylglycerol to form PC. PC can also be transformed from PE directly. PEs account for 15–25% of total phospholipids in mammalian cells (Vander Veen et al., 2017). PE is derived from the synthesis of diacylglycerol and CDP-ethanolamine. The decreased ratio of PC to PE, observed in NAFLD patients, could impair the permeability of cellular membrane and induce damage to hepatocytes (Li et al., 2006). Moreover, modulating the balance of PC and PE has shown beneficial effects on NAFLD (Calzada et al., 2016). Studies have demonstrated that orally treatment of *Lactobacillus*-containing probiotic formulation could increase the levels of PC in HFD-induce obese rat model (Shin et al., 2018). Negative correlations of PC (18:3), PC (20:2) and *Lachnoclostridium* have been found in obese mice (Liu et al., 2018). Our spearman’s analysis also showed a positive correlation between *Lactobacillus* and PC level, and negative correlations of *Lachnoclostridium, Enterococcus* and PC. Additionally, we observed a negative correlation between PE and *Romboutsia*. Given these correlations, its likely the modulatory effects of DCH on glycerophospholipid metabolism might occur through affecting the abundances of *Lactobacillus, Lachnoclostridium* and *Romboutsia*.

### Glycine/Serine/Threonine Metabolism

The dysfunction of glycine, serine and threonine metabolism has also been associated with the NAFLD-related metabolic disorders (Huang et al., 2018). Increased levels of glycine and decreased levels of L-threonine were observed in NAFLD model rats, with DCH treatment reversing the trend. L-threonine is an essential amino acid in mammal cells and has shown beneficial effects on NAFLD through inducing the synthesis of phospholipid and oxidation of fatty acids (Zhang et al., 2020). Unlike other amino acids, L-threonine can be transformed into different amino acids, such as glycine, through specific enzymatic reaction rather than through the catalysis of dehydrogenase or transamination. Glycine can be transformed into serine, which can be subsequently transformed into pyruvate through dehydration and deamination. Pyruvate can then enter the citrate cycle to generate ATP. Clinical studies showed an increase of plasma glycine levels in patients with NAFLD (Melania et al., 2018). Excessive glycine levels can disrupt the metabolism of amino acids and the citrate cycle. Spearman’s correlation analysis results indicated that *Lactobacillus, Romboutsia*, and *Bacteroides* exhibited positive correlations with L-threonine, while *Akkermansia*, *Enterococcus, Enterobacteriaceae,* and *Lachnoclostridium* exhibited negative correlations. Likewise, positive corelations have been demonstrated of *Lactobacillus, Romboutsia* and L-threonine in mice after fed with *Ganoderma lucidum* spores oil (Wu et al., 2020). No bacteria examined showed significant correlations with glycine. The changes in glycine levels after DCH treatment might be caused by the transformation of L-threonine.

## Conclusion

**FIGURE 5:**
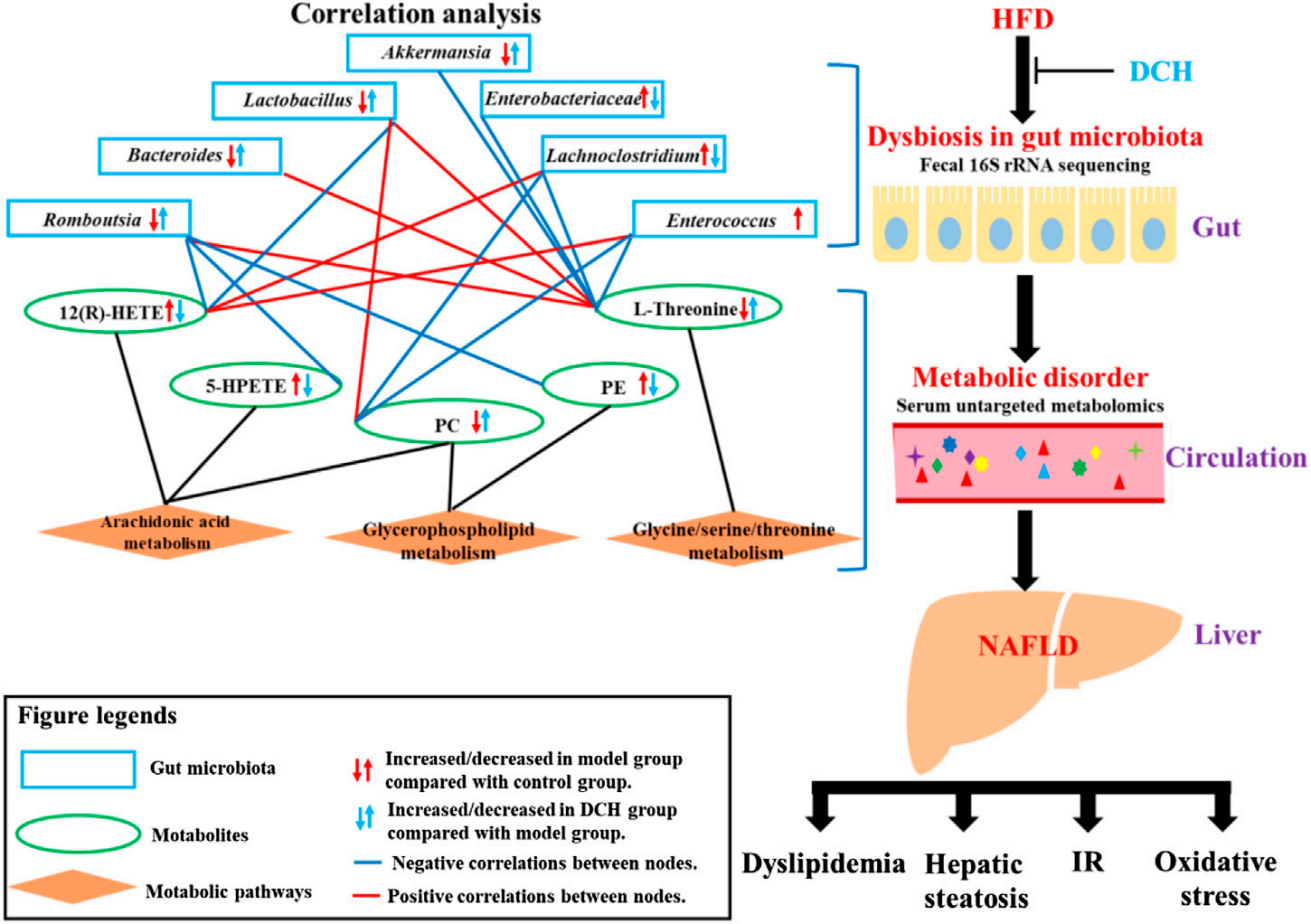
Schematic diagram of DCH’s working mechanisms on NAFLD.

Overall, our study revealed the various ameliorative effects of DCH on NAFLD, including reducing the hepatic steaosis, improving dyslipidemia and IR, and enhancing the liver’s anti-oxidative abilities. The mechanisms of DCH on NAFLD were likely linked to the improvement of the dysbiosis of gut microbiota and modulation of AA, glycerophospholipid, and glycine/serine/threonine metabolism (Figure 5). However, the detailed modulatory effects of DCH on gut microbiota and serum metabolism should be further verified using metagenomics and secondary mass spectrometry in metabolomics. Recent studies have also developed a gut microbiota depletion model using antibiotics to further verify the modulatory effects of drugs on gut microbiota (Cui et al., 2018). Fecal transplantation from drug-treated groups to experimental model groups has also been used as a novel approach to validate modulatory effects of drugs on gut microbiota (Li et al., 2018). Gut microbiota depletion and fecal transplantation could be used in future studies to validate DCH’s ability to modulate the serum metabolism through improving the dysfunction of gut microbiota community. Despite the need for further specific investigation, our study has illuminated the mechanisms of DCH on NAFLD patients, and highlighted the utility of combination 16S rRNA sequencing-metabolomics to investigate the mechanisms of gut microbiota and host metabolism in Chinese herbal formulas.

## Data Availability Statement

The sequencing data in our study has been uploaded in the BioProject: PRJEB40601, https://www.ncbi.nlm.nih.gov/bioproject/PRJEB40601/.

## Ethics Statement

The animal study was reviewed and approved by Animal Ethics Committee at Tianjin University of Traditional Chinese Medicine.

## Author Contributions

HC wrote the manuscript. HC, YL, LJ, LY, and YW conducted animal experiments. HC, LW, JL, LY, and HSW finished molecular bioassays. ZZ, HWW, XL, and YP provided technical guidance for the whole work. All authors contributed to the article and approved the submitted version.

## Funding

This work was supported by National Science Foundation of China (81703828), Natural Science Foundation of Tianjin (17JCYBJC42800), Science and Technology Projects in Key Fields of Traditional Chinese Medicine of Tianjin Municipal Health Commission (No. 2020006) and Public Welfare Research Projects in Jiaxing (SQ2018001355).

## Conflict of Interest

The authors declare that the research was conducted in the absence of any commercial or financial relationships that could be construed as a potential conflict of interest.
