# Supplementary material for: Da-Chai-Hu Decoction Ameliorates High Fat Diet-Induced Nonalcoholic Fatty Liver Disease Through Remodeling the Gut Microbiota and Modulating the Serum Metabolism
Source: Front Pharmacol. 2020 Nov 27;11:584090. doi: 10.3389/fphar.2020.584090 (PMC7732620; doi:10.3389/fphar.2020.584090)
Supplement: Supplementary file 1 [file datasheet1.docx]

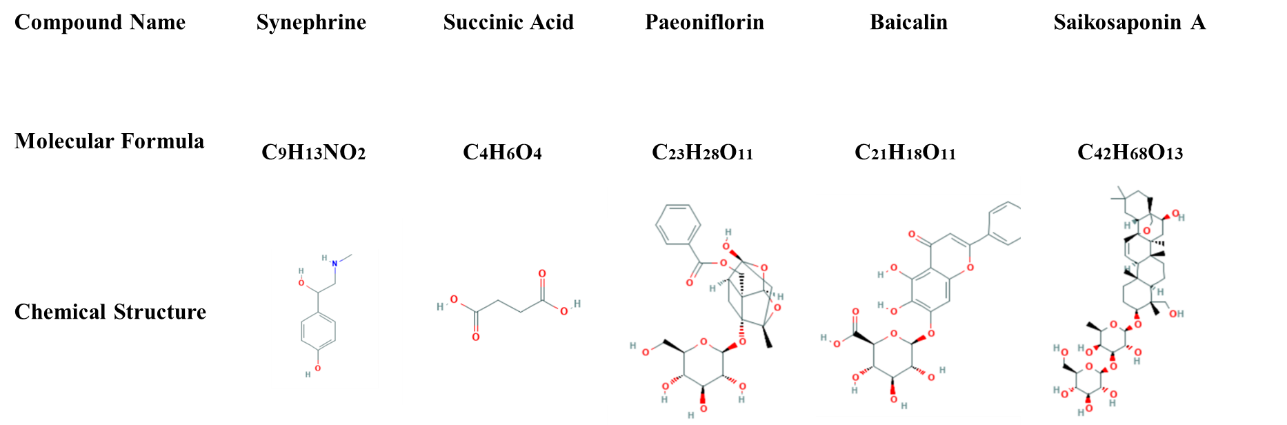


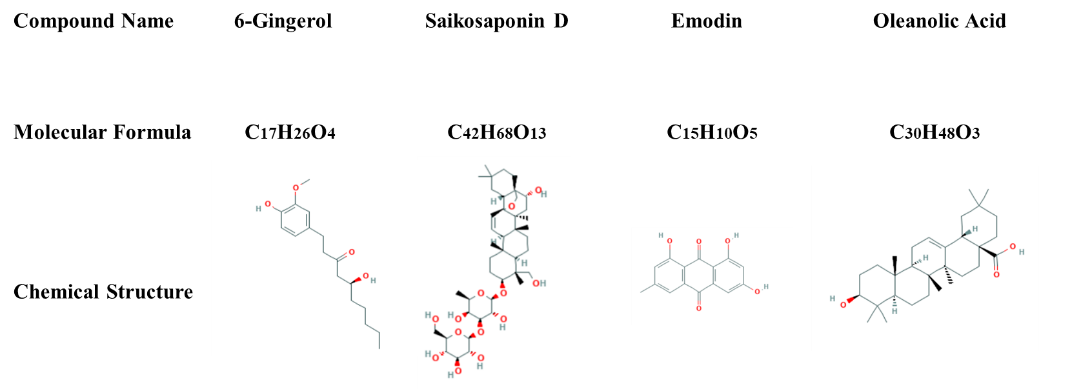


**Figure S1:** The molecular formulas and chemical structures of reference standards.


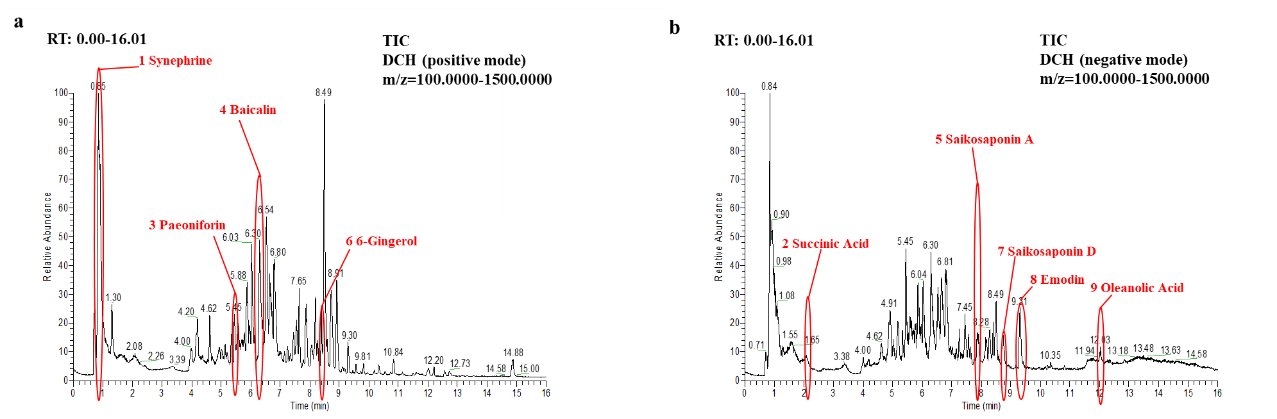


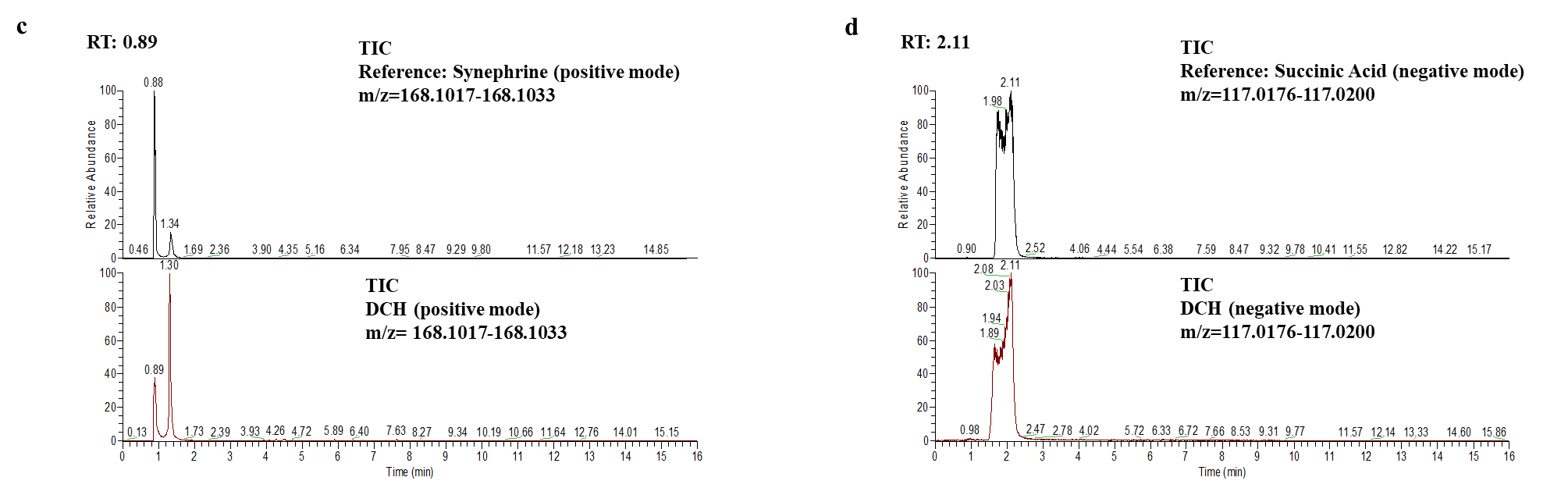


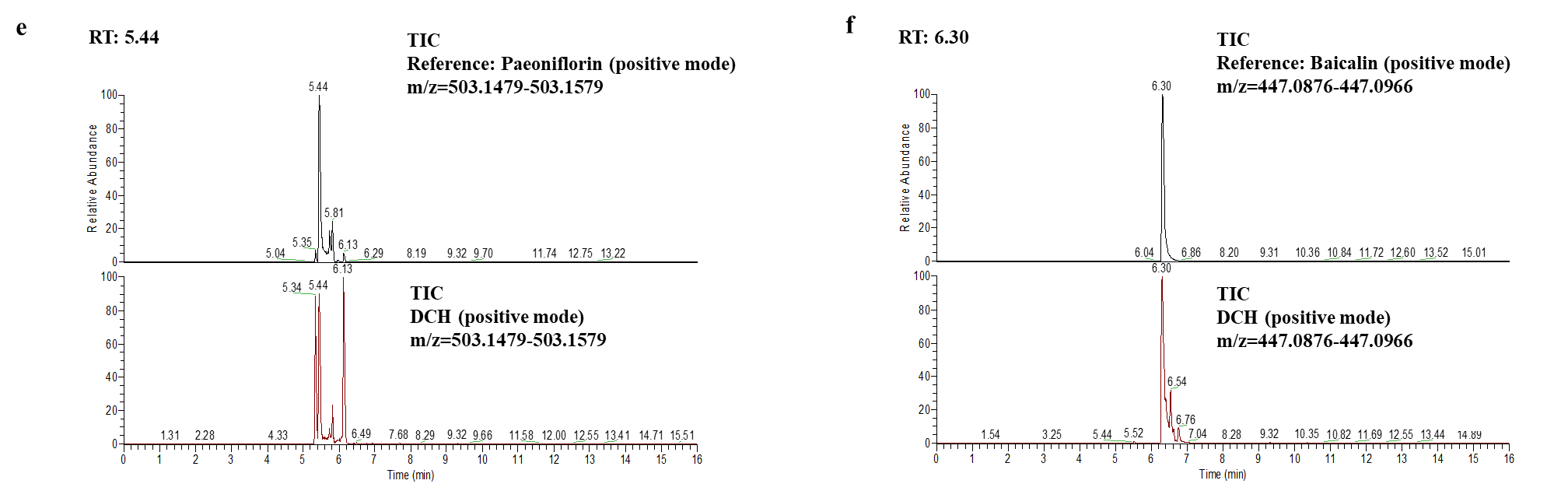


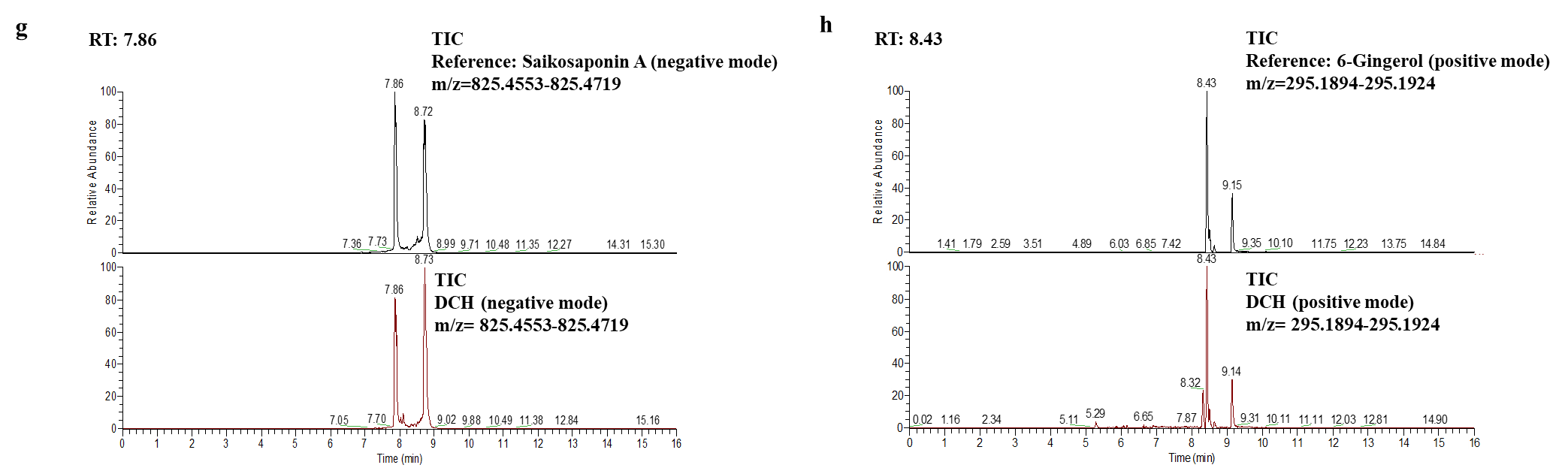


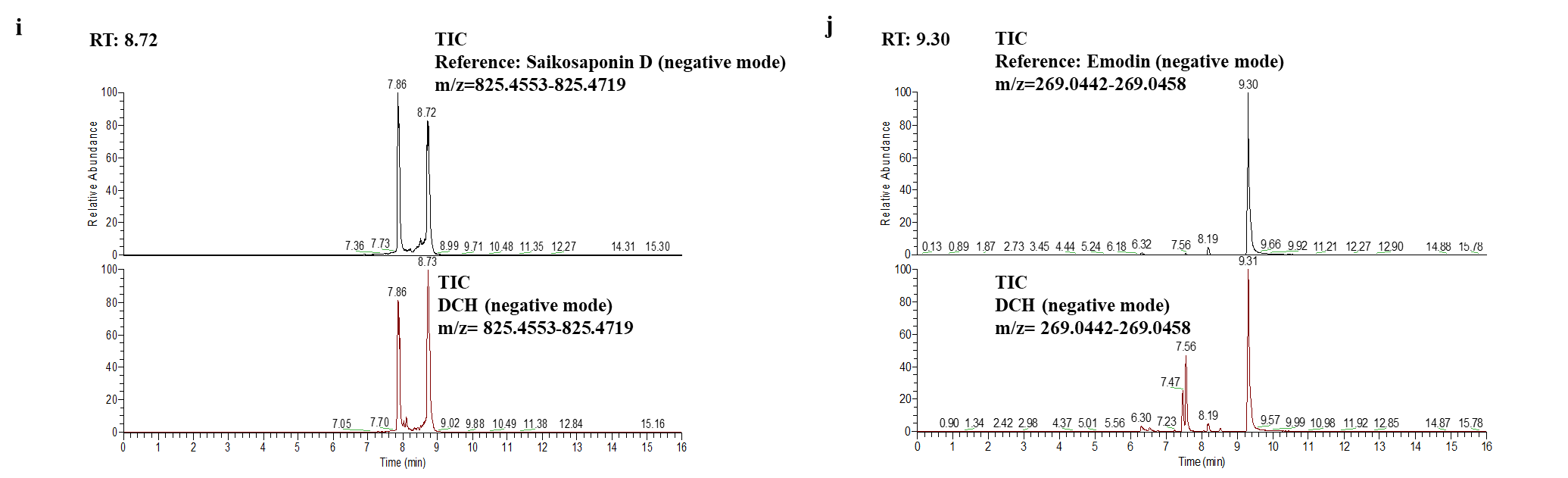


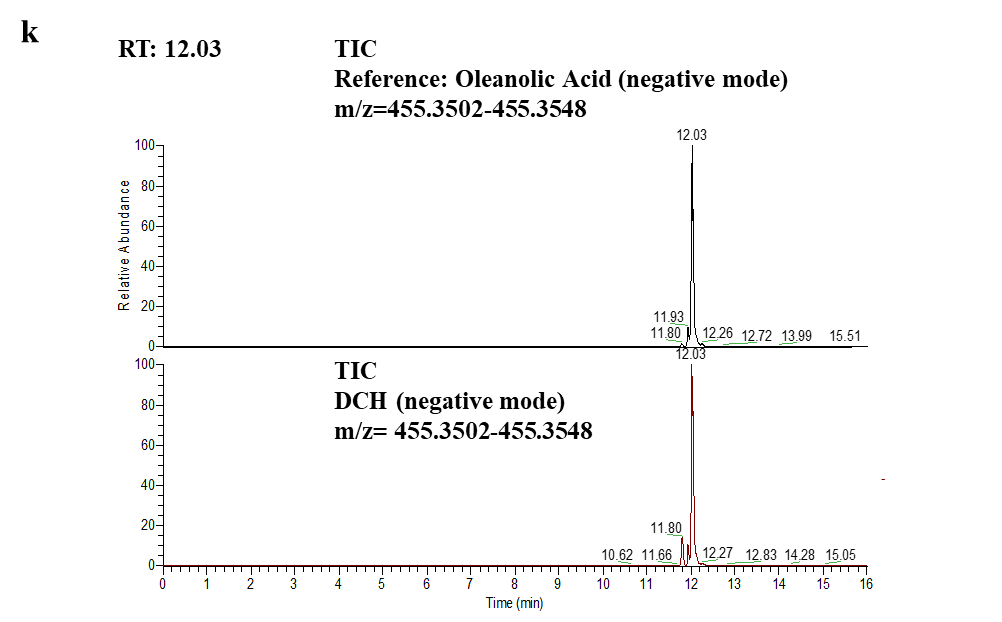


**Figure S2:** The chemical profiles of DCH using HPLC-MS. (**a, b**) The total ion chromatogram (TIC) in positive (**a**) and negative ion modes (**b**). (**c-k**) The main bioactive compounds of synephrine (**c**), succinic acid (**d**), paeoniflorin (**e**), baicalin (**f**), saikosaponin A (**g**), 6-gingerol (**h**), saikosaponin D (**i**), emodin (**j**) and oleanolic acid (**k**). The bioactive compounds detected in DCH were in red peaks and were confirmed by the reference standards in black peaks.

**TABLE S1 The characteristic fragment ions of reference standards in DCH**

| **Marking**  **peak no.** | **Name** | **RT**  **(min)** | **Ion** | **m/z** | **Herbs** |
| --- | --- | --- | --- | --- | --- |
| 1 | Synephrine | 0.89 | [M+H]^+^ | 168.1025 | *Citrus* × *aurantium* L. |
| 2 | Succinic Acid | 2.11 | [M-H]^-^ | 117.0188 | *Pinellia ternata* (Thunb.) Makino |
| 3 | Paeoniforin | 5.44 | [M+Na]^+^ | 503.1529 | *Paeonia lactiflora* Pall. |
| 4 | Baicalin | 6.30 | [M+H]^+^ | 447.0921 | *Scutellariae baicalensis* Georgi |
| 5 | Saikosaponin A | 7.86 | [M-H]^-^ | 779.4582 | *Bupleurum chinense* DC. |
| 6 | 6-Gingerol | 8.43 | [M+H]^+^ | 295.1909 | *Zingiber officinale* Roscoe |
| 7 | Saikosaponin D | 8.72 | [M-H]^-^ | 779.4582 | *Bupleurum chinense* DC. |
| 8 | Emodin | 9.30 | [M-H]^-^ | 269.0450 | *Rheum officinale* Baill. |
| 9 | Oleanolic Acid | 12.03 | [M-H]^-^ | 455.3525 | *Ziziphus jujuba* Mill. |
